# Risk factors for revision of Birmingham Hip Resurfacing arthroplasty. A single-center study with a median 13-year follow-up

**DOI:** 10.1016/j.jor.2025.07.013

**Published:** 2025-07-14

**Authors:** Alexander Oxblom, Håkan Hedlund, Li Felländer-Tsai, Ola Rolfson, Harald Brismar

**Affiliations:** a Divison of Orthopaedics and Biotechnology, CLINTEC, Karolinska Institutet, H9 Klinisk vetenskap, intervention och teknik, H9 CLINTEC Ortopedi och bioteknologi, 141 52 Huddinge, Sweden; b Department of Orthopaedics, VO KOU, Södertalje Hospital, 152 86, Södertalje, Sweden; c Department of Orthopaedics, Visby Hospital, S:t Göransgatan 5, 621 55, Visby, Sweden; d Department of Reconstructive Orthopaedics, Karolinska University Hospital, 141 86, Stockholm, Sweden; e Department of Orthopaedics, Institute of Clinical Sciences, Sahlgrenska Academy, University of Gothenburg, Göteborgsvägen 31, R-huset, plan 7, 431 80 Mölndal, Sweden

**Keywords:** Metal-on-metal hip resurfacing, Total hip arthroplasty, Revision hip arthroplasty

## Abstract

**Background:**

Consensus is lacking regarding follow-up after metal-on-metal hip resurfacing (MoM-HR). This study examines risk factors for revision and evaluates the need for sequential follow-up.

**Patients and methods:**

288 consecutive patients operated on with a unilateral Birmingham Hip Resurfacing (BHR) 2001–2014, having at least one x-ray and one metal ion sampling, were followed until 2022. Hazard ratios (HR) of revision were calculated for sex, age, femoral component head size, implant positioning, serum cobalt (sCo), and serum chrome (sCr) concentrations. The relative risk (RR) of revision if risk factors were present was calculated. Radiologic changes in component positioning were analyzed in 288 patients, and metal ion changes in 147 patients.

**Results:**

The median follow-up was 13 years. Factors associated with revision were sCo and/or sCr >5 μg/l at first follow-up, a post-operative anteversion <5 or >25°, and femoral head component size <50 mm. Patients with one or more risk factors at first follow-up had a RR of 10 (95 % CI 2.5–42) to be revised compared to those without risk factors. A ≥10° change in the stem shaft angle was associated with an increased OR of revision (OR 14, 95 % CI 4.4–43). A change in sCo and/or sCr from ≤5 to >5 μg/l between follow-ups was associated with an increased risk of revision (OR 8.5, 95 % CI 1.3–55).

**Conclusions:**

Patients with risk factors at first follow-up, a change from serum metal ions ≤5 to >5 μg/l, and a ≥10° change in the stem shaft angle at sequential follow-ups warrant continuous follow-up even if asymptomatic.

## 1 Introduction

Metal-on-metal hip resurfacing (MoM-HR) gained popularity in the first decade of the 21st century as it was thought to provide a durable surface minimizing complications due to polyethylene wear, anatomic hip restoration allowing a greater range of motion, and larger femoral head size reducing risk of dislocation.^1^^,^^2^ The use of MoM-HR peaked around 2007 in Sweden.^3^ The National Joint Registry (NJR) reported that MoM-HR was used in about 11 % of all primary hip arthroplasties in 2006 with a decrease to <1 % in 2022.^4^

The cobalt-chrome alloy (CoCr) articulation in MoM-HR may cause metal wear resulting in an outflow of cobalt and chrome particles locally into the periprosthetic tissues, potentially causing adverse tissue reactions, and systemically into the bloodstream.^5^^,^^6^ MoM-HR is not recommended in patients ≥55 years due to the increased likelihood of poor bone stock and excellent results with conventional total hip arthroplasty (THA).^2^ Moreover women are reported to have a higher risk for revision surgery as well as patients with prior childhood hip disorders and the need for a femoral head component size.^2^ Local tissue reaction complications, the uncertainty of systemic impact of long-term increased metal ion levels, and the higher revision risk of MoM-HR compared to conventional THAs have decreased the use of hip resurfacing worldwide.^7^

Most follow-up protocols of MoM-HR include consecutive hip x-ray imaging and serum or blood metal concentration analysis but there is no consensus regarding the extent of follow-up.

The primary aim of this study was to identify factors, available at mean follow-up of 5 years, associated with revision following primary MoM-HR with a Birmingham Hip Resurfacing (BHR). The secondary aims were to investigate the association between changes in measures of implant positioning and metal ions and revision.

## 2 Patients and methods

### 2.1 Patients and setting

The study population consisted of 410 consecutive patients operated on with a BHR (Smith & Nephew, Andover, Massachusetts, USA) at Karolinska University Hospital in Huddinge. All patients were operated on in 2001–2014 by two experienced hip arthroplasty surgeons using a posterior approach. The study period was 2001–2022, and all patients were followed up until the end of 2022. Data were retrieved from electronic patient records (CGMtakecare ®, CompuGroup Medical, Solna, Sweden) and the Swedish Arthroplasty Register. The study was approved by the Regional Ethical Review Board in Stockholm (2017/1841-31/2).

**Fig. 1:**
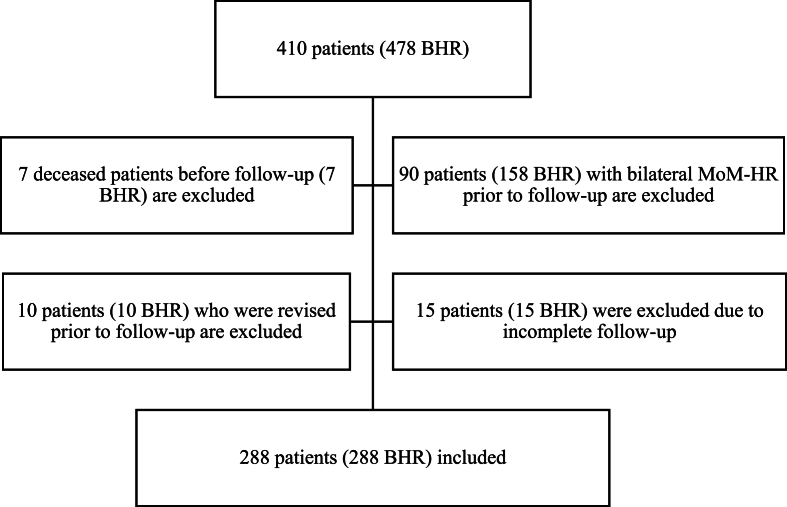
Flowcharts of studied patients.

The inclusion criteria were an unrevised BHR and a contralateral hip not operated on with a MoM-HR. Only patients who had a complete follow-up, consisting of serum cobalt (sCo) and serum chrome (sCr) analysis combined with one postoperative pelvic and true lateral hip x-rays, were included. Patients who had incomplete follow-up (n = 15), had deceased (n = 7), had received bilateral MoM-HR (n = 90), or had been revised (n = 10) without prior complete follow-up were excluded (Fig. 1). The first serum metal ions were retrieved in 2011, and from 2014 the intention was to follow up patients every 5th year with x-ray examination and serum metal ion analysis. More frequent investigations were offered to those with either abnormal findings on X-rays or sCo and/or sCr (sCo–sCr) concentration >5 μg/l 131 patients had only one metal ion follow-up. Changes in serum metal ion concentrations between the first and last available serum samples were analyzed in 147 patients.

### 2.2 Outcome measures

Samples of sCo and sCr were collected with the first vial discarded to avoid metal contamination from the needle. After coagulation at room temperature, the blood was centrifuged to separate the serum. The samples were analyzed at ALS Scandinavia, Luleå, Sweden, with mass spectrometry using ICP-SFMS technology.

Using the “Ortho Toolbox” instrument on a SECTRA workstation (PACS, Sectra AB, Linköping, Sweden), three radiographic measurements were performed. First, the inclination angle was determined by measuring the angle formed between the ischial tuberosity line and a line drawn between the two farthest points along the AP elliptic projection of the cup. Then the cup version angle, built up by the line connecting the outermost edges of the cup and a line drawn perpendicular to the table, was measured on a cross-table lateral radiograph.^8^ Lastly, the stem shaft angel (SSA) was ascertained from the AP pelvic view, measuring the angle formed between a line drawn along the center of the femoral shaft and a line drawn along the center of the peg of the femoral component.^9^ Both the anteversion angle and the inclination angle were categorized into groups according to Lewinnek's safe zones^10^: anteversion <5°, anteversion 5–25° (optimal positioning), anteversion >25°, inclination <30°, inclination 30–50° (optimal positioning), and inclination >30°. We also categorized the SSA into 3 groups: <120°, 120–140° (optimal positioning based on normal femur anatomy),^11^ and >140°. Serum metal ion concentrations were categorized as either ≤5 μg/l if both sCo and sCr were below the limit or >5 μg/l if either sCo or sCr or both were >5 μg/l.^12^ All radiographic measurements were performed by the first author (AO).

Sex, age, and size of the implanted femoral resurfacing head component were also studied. Primary diagnosis was noted, but not further investigated in the analysis. Demographics are presented in Table 1.

**Table 1:** Patient demographics.

Characteristics	Level	All
**Number of patients**		288
**Patient sex, n (%)**	Woman	69 (24)
	Man	219 (76)
**Mean age at primary surgery, years (range)**		51 (27–71)
**Median year of surgery (IQR)**		2009 (2006, 2011)
**Median femoral head component size (IQR)**		52 (48, 54)
**Femoral head component size** < **50 mm, n (%)**	Yes	74 (26)
	No	214 (74)
**Preoperative diagnosis, number (%)**	Primary coxarthrosis	233 (81)
	Dysplastic coxarthrosis	36 (13)
	Secondary coxarthrosis	9 (3)
	Osteonecrosis	6 (2)
	Rheumatoid arthritis	3 (1)
	Posttraumatic arthrosis	1 (<1)
**Postoperative inclination angle in degrees, cohort (%)**	30–50°	251 (87)
	<30°	12 (4)
	>50°	25 (9)
		
**Postoperative anteversion angle in degrees, cohort (%)**	5–25°	167 (58)
	<5°	74 (26)
	>25°	47 (16)
		
**Postoperative stem shaft angle in degrees, cohort (%)**	120–140°	210 (73)
	<120°	2 (1)
	>140°	76 (26)
**Mean time until serum metal analysis, yrs (range)**		6.8 (0.80–17)
**Median serum cobalt concentrations, μg/l (IQR)**		1.2 (0.88, 1.9)
**Median serum chrome concentrations, μg/l (IQR)**		2.1 (1.4, 3.3)
**Serum cobalt and/or chrome concentration** > **5 μg/l, n (%)**	Yes	42 (15)
	No	246 (85)

IQR, Interquartile range.

### 2.3 Data analyses

The hazard ratio (HR) of revision was analyzed separately for each variable using univariate Cox proportional hazards regression analysis. The postoperative radiographic measurements and the first serum metal ion analysis were used in the analysis. The index year (T = 0) was the year of the first serum metal collection. Factors with a 95 % confidence interval not encompassing 1 were included in the multivariate Cox regression analysis to adjust for the effect of the different variables. Based on the results from our univariate Cox regression analysis, we defined a “suboptimal” and “optimal” group to calculate the relative risk (RR) of revision. Logistic regression analysis was used to analyze the odds ratio (OR) of revision depending on radiological changes in angle measurements between the postoperative x-ray and the last follow-up. Logistic regression analysis was also performed to analyze the OR of revision depending on changes in serum cobalt and chrome concentrations between the first and last follow-up. The software STATA version 16.0 (StataCorp LLC., College Station, Texas, United States) was used for statistical analyses.

## 3 Results

The median follow-up time was 13 years (IQR, 11; 16), and 32 patients (11 %) were revised. The mean age at primary surgery was 51 years (range, 27 to 71), and 24 % were women. The mean time until first serum metal collection was 6.8 years (range, 0.8 to 17), and the median sCo and sCr were 1.2 (IQR, 0.88; 1.9) and 2.1 (IQR, 1.4; 3.3) respectively (Table 1). sCo–sCr >5 μg/l was found in 42 patients (15 %).

### 3.1 Risk factors of revision

When Cox proportional hazard models were applied to each covariate individually, anteversion <5° (compared to 5–25° of anteversion), anteversion >25° (compared to 5–25° of anteversion), femoral head component size <50 mm, and sCo–sCr >5 μg/l were associated with revision (Table 2). When adjusting for these four variables in a multivariate model, sCo–sCr >5 μg/l (HR 3.5, 95 % CI 1.6, 7.8), anteversion <5° (HR 3.3, 95 % CI 1.3, 8.3), anteversion >25° (HR 3.7, 95 % CI 1.4, 10), and femoral head component size <50 mm (HR 2.2, 95 % CI 1.1, 4.5) were associated with revision.

**Table 2:** Univariate and multivariate Cox proportional hazard regression analysis of the hazard ratio of revision surgery. The reference group consisted of men, femoral head component size >48 mm, cup inclination angle 30–50°, cup anteversion angle 5–25°, stem shaft angle 120–140°, and serum cobalt and chrome concentrations ≤5 μg/l.

	Cohort	Crude HR (95 % CI)	Adjusted HR (95 % CI)
Age		1.0 (0.98, 1.1)	NA
Sex (woman)		1.9 (0.95, 3.9)	NA
Femoral head component size <50 mm		2.3 (1.2, 4.7)	2.2 (1.1, 4.5)
Inclination angle	<30°	1.8 (0.43, 7.7)	NA
	>50°	2.1 (0.80, 5.4)	NA
Anteversion angle	<5°	5.1 (2.2, 11.8)	3.3 (1.3, 8.3)
	>25°	3.8 (1.4, 10.3)	3.7 (1.4, 10.0)
Stem shaft angle	>140°	0.79 (0.34, 1.8)	NA
Serum metal concentrations >5 μg/l		5.2 (2.6, 10.5)	3.5 (1.6, 7.8)

HR, Hazard ratio; 95 % CI, 95 % confidence interval; NA, not applicable.

In the “optimal” group, consisting of patients having a femoral head implant diameter ≥50 mm, a 5–25° postoperative cup anteversion angle, and sCo and sCr concentration ≤5 μg/l at the first follow-up, 2 of 117 (2 %) were revised. In the “suboptimal” group, consisting of patients with anteversion outside the interval 5–25°, and/or femoral head implant diameter <50 mm, and/or sCo–sCr concentration >5 μg/l at the first follow-up, 30 of 171 (18 %) were revised (RR 10, 95 % CI 2.5, 42; p < 0.001).

### 3.2 Radiologic changes

The simple logistic regression analysis of the association between significant radiologic positioning changes (≥10°) from postoperative to last plain x-ray control, median 9 years (IQR, 6; 12), revealed that a ≥10° change in stem-shaft angle was associated with increased OR of revision surgery (OR 14, 95 % CI 4.4, 43) (Table 3).

**Table 3:** Simple logistic regression analysis of the odds ratio of revision depending on changes in radiological angle measurements ≥10° between postoperative x-ray and last radiologic follow-up compared to those with a <10° change.

Postoperative to last follow-up	Number of patients (%) (n = 288)	Crude OR (95 % CI)
**≥10° change**		
Anteversion angle	31 (11)	0.84 (0.24, 2.9)
Inclination angle	1 (<1)	NA
Stem shaft angle	14 (5)	14 (4.4, 43)

OR, odds ratio; 95 % CI, 95 % confidence interval; NA, not applicable.

### 3.3 Changes in serum metal concentrations

More than one serum metal ion analysis was performed for 147 patients. The median time between the first and last metal ion analysis was 4.4 years (IQR, 2.7; 5.8). sCo and sCr remained ≤5 μg/l in 111 patients between first and last follow-up, 5 of those were later revised. sCo–sCr increased between follow-up from ≤5 μg/l to >5 μg/l in 7 patients, 2 of whom were later revised. Of the 29 patients who initially had sCo–sCr >5 μg/l, 9 were later revised. Both an increase in metals from sCo and sCr ≤5 μg/l to concentrations >5 μg/l and an initial sCo–sCr >5 μg/l were associated with increased OR of revision (OR 8.5, 95 % CI 1.3, 55 and OR 9.5, 95 % CI 2.9, 31 respectively) (Table 4).

**Table 4:** Simple logistic regression analysis of the odds ratio of revision. The reference group consisted of 111 patients with serum metal concentrations ≤5 μg/l on both the first and last serum metal collection.

	Number of patients (%) (n = 147)	Crude OR (95 % CI)
Serum metal concentrations from ≤5 μg/l to >5 μg/l	7 (5)	8.5 (1.3, 55)
Serum metal concentrations from >5 μg/l to either >5 μg/l or ≤5 μg/l	29 (20)	9.5 (2.9, 31)

OR, odds ratio; 95 % CI, 95 % confidence interval.

## 4 Discussion

Excessive cup anteversion, insufficient cup anteversion, small femoral head component size, and sCo–sCr >5 μg/l were associated with an increased HR of revision. Patients who had one or more risk factors at the first follow-up were 10 times more likely to be revised than those without risk factors. A ≥10° change in stem-shaft angle was associated with increased risk of revision surgery. Patients with initial serum metal concentrations ≤5 μg/l who increased to sCo–sCr >5 μg/l at later follow-up had an increased risk of revision compared to those without an increase. However, only 7 of 118 patients with initial sCo and sCr ≤5 μg/l increased to >5 μg/l.

In accordance with previous studies, we found a higher risk for revision if the acetabular cup was not positioned within the anteversion safe-zone.^13^ Contrary to a previous study,^14^ we could not find a statistically significant increased risk of revision in patients with suboptimal cup inclination. This may be explained by the fact that there were relatively few patients whose acetabular cups were positioned outside the inclination safe-zone interval. Studies of wear characteristics of the CoCr alloy of MoM-HR have shown that the design is prone to edge loading in steeply inclined acetabular cups or improperly anteverted cups.^15^ Edge loading describes the wear when the loaded surface of the femoral head component comes into contact with the edge of the acetabular component. In a previous study on the current patient cohort, we found insufficient anteversion to be a major factor associated with increased metal ion levels.^16^ Therefore, correct cup positioning seems to be of utmost importance to prevent excessive wear and increased serum metal concentrations and to reduce the risk of revision.

Small femoral head size was associated with a higher risk of revision surgery in line with previous reports but contrary to others we did not find women to have increased risk of revision.^17^ The fact that women have smaller femoral head sizes probably explains why a higher proportion of women are revised, i.e. women with larger head sizes are not more likely to be revised than men with comparable head sizes.

A sCo–sCr >5 μg/l at first follow-up and an increase from ≤5 μg/l to >5 μg/l were associated with revision. The relationship between elevated metals and revision is well studied, and this is probably due to the local toxic effects of the metal wear on the tissues surrounding the joint, leading to osteolysis and pseudotumor formation.^18^

Patients who have one or more risk factors (serum metals >5 μg/l, anteversion outside 5–25°, and femoral head component size <50 mm) were 10 times more likely to undergo revision surgery than those without any risk factors. Thus, avoiding these risk factors is essential to optimize surgery results. Given the almost 3–fold increased risk of revision for smaller head sizes, a BHR should not be considered unless a cup >48 mm can be fitted. An optimized cup positioning is also crucial. Since the accuracy of correct cup positioning is low even among high-volume surgeons,^19^ navigation technology may be needed for increased precision.^20^

Several national agencies recommend long-term regular follow-up, including hip x-rays and serum or blood metal ion concentrations of asymptomatic patients with hip resurfacing prostheses.^21^^,^^22^ Contrary, the Food and Drug Administration (FDA) has not found sufficient evidence for regular testing of metal ion concentrations in their updated recommendations if the patient is asymptomatic.^23^ Our results show that it is uncommon that serum metal levels change substantially between measurements 5–10 years postoperatively. Most patients who have initial sCo and sCr ≤5 μg/l remain ≤5 μg/l at follow-up, and those who have initially high levels stay high at follow-up. This suggests that patients who have low metal ion levels and no other risk factors at a mean 7-year follow-up do not need to be followed closely if asymptomatic, considering the low probability of an increase in metal ion levels. It is, however, to be noted that a repeat metal ion concentration increasing from a level ≤5 to >5 μg/l is a risk factor for revision. On the contrary, patients who have one or more risk factors at first follow-up, including elevated serum metal ion levels, need closer monitoring since their risk of revision is substantially elevated.

In this study, sequential monitoring of cup implants did not reveal changes in position, i.e., most cup implants were stable. The ≥10-degree change in anteversion between follow-ups, seen in 10 % of the patients (Table 3), could relate to measuring errors due to difficulties reproducing identical positioning at sequential x-rays in lateral decubitus position. On the other hand, a change of the femoral head component position, the SSA, seems to be a factor related to revision. This change in position is probably due to femoral head necrosis and loss of component bone support. Sequential X-rays may thus be of importance.

There are some potential limitations to our study. The assessment of implant positioning on plain X-rays is subject to variability due to difficulties in positioning the patients. This especially affects measurements of the cup version on true lateral views, which may increase the risk of making a Type 2 error. Another weakness is that we have not analyzed the contact patch-to-rim distance (CPRD), an important measurement to determine the risk of excessive MoM-HR wear.^24^ A small CPRD increases the risk of wear, which probably explains why smaller femoral head components are associated with an increased risk of revision surgery. However, CPRD is challenging to calculate on plain x-rays and is more precisely measured with CT. We did not have CTs on a majority of patients, and we believe standard X-rays to be more justifiable in the clinical situation, also taking the radiation dose and costs into account. Moreover, analyzing one brand of MoM-HR affects the generalizability of our study. The majority of patients had either been referred to our institution or contacted us directly with an interest in receiving a MoM-HR. Our population is therefore not representative of the average THA population. Since our focus was on analyses of risk factors for revision after BHR and to determine a more appropriate follow-up regime, such bias does not seem important. Also, the first metal ion analysis was performed at a mean of 7 years, constituting a problem of not following our patients coherently. This was because metal ion analysis collection was not a part of national follow-up guidelines at the beginning of our study period, resulting in the first patient being operated on in 2001, but the first metal ion analysis was collected in 2011. Additionally, 122 patients (30 %) were excluded from the analysis since they had received a contralateral MoM-HR before follow-up (90 patients) or had been revised before follow-up (10 patients). It could be argued that the loss of the 10 revised patients could affect our results.

## 5 Conclusion

Incorrect anteversion angle of the cup on postoperative x-ray, femoral head component size <50 mm, and serum metal concentrations >5 μg/l were associated with a higher risk of revision surgery in patients operated on with BHR arthroplasty. Patients without risk factors were unlikely to undergo future revision surgery, while patients with risk factors warrant closer follow-up. Even if patients with low serum metal ion levels at initial follow-up are unlikely to increase in serum metal concentration to levels >5 μg/l at sequential follow-up, those who do have an increased risk of revision. Our results also suggest that there is an increased risk of revision when SSA changes substantially between postoperative imaging and follow-up.

## CRediT authorship contribution statement

**Alexander Oxblom:** Conceptualization, Data curation, Formal analysis, Investigation, Methodology, Validation, Visualization, Writing – original draft, Writing – review & editing. **Håkan Hedlund:** Data curation, Formal analysis, Investigation, Methodology, Validation, Visualization, Writing – original draft, Writing – review & editing. **Li Felländer-Tsai:** Conceptualization, Data curation, Formal analysis, Funding acquisition, Investigation, Methodology, Validation, Visualization, Writing – original draft, Writing – review & editing. **Ola Rolfson:** Data curation, Formal analysis, Funding acquisition, Investigation, Methodology, Project administration, Supervision, Validation, Visualization, Writing – original draft, Writing – review & editing. **Harald Brismar:** Conceptualization, Data curation, Formal analysis, Investigation, Methodology, Validation, Visualization, Writing – original draft, Writing – review & editing.

## Patient consent

The data used for this manuscript was collected as a part of the department's clinical follow-up and not primarily collected because of the research. Therefor no specific patient consent was obtained regarding the research.

## Ethical statement

The study was approved by the Regional Ethical Review Board in Stockholm (2017/1841-31/2).

## Funding

This study was supported by research grants from the Stockholm County Council (dnr RS 2019-1054, 20200305).

## Declaration of conflicting interests

The authors declared no potential conflicts of interest with respect to the research, authorship, and/or publication of this article.
